# Identification of fungi associated with soybeans and effective seed disinfection treatments

**DOI:** 10.1002/fsn3.1166

**Published:** 2019-08-22

**Authors:** Diana Escamilla, Maria Luciana Rosso, Bo Zhang

**Affiliations:** ^1^ Department of Agronomy Purdue University West Lafayette IN USA; ^2^ School of Plant and Environmental Sciences Virginia Polytechnic Institute and State University Blacksburg VA USA

**Keywords:** disinfection treatments, fungi, seed‐borne, soybean sprout

## Abstract

Sprouts can be a vehicle for the transmission of several pathogens capable of causing human illness, and the potential source of contamination is seed used for sprouting. The limited information about seed‐borne pathogens as well as their incidence on soybean seeds for soybean sprout industry led the objectives of this study that were to identify seed‐borne pathogens on commercial sprout soybean seeds and to evaluate different decontamination treatments on disinfection effectiveness and sprout quality. Seeds of “MFS‐561,” a sprout soybean cultivar, from three production regions were used in this study. The internal transcribed spacer (ITS1 and ITS2) DNA sequences of the isolated fungi from MFS‐561 seeds were used for species identification. Seven disinfection treatments were evaluated on their effectiveness on reducing fungal incidence and impact on sprout characteristics. Out of 55 fungal isolates obtained from the soybean seeds, seven species and six genera were identified. The most frequent genera across regions were *Alternaria*, *Diaphorte,* and *Fusarium.* The treatment of soaking seeds in 2% calcium hypochlorite for 10 min and 5% acetic acid for 2 min before sprouting were promising seed disinfection treatments as they significantly reduced fungi incidence without any negative effects on sprout quality.

## 1 INTRODUCTION

Sprout soybeans, a soyfood made from small seeded varieties (<12 g/100 seeds), is an important vegetable consumed in many Asian countries such as Korea, China, and Japan. In Korea, more than 500,000 tons of soybean sprouts is consumed annually as a vegetable in soups, salads, and side dishes (Hwang, Jeong, & Lee, 2004; Lee, Shannon, Jeong, Lee, & Hwang, 2007; Zhang et al., 2010). In the united states, 10% of the population eats sprouts (alfalfa, mung bean, and soybean) regularly, resulting in a $250 million market (Sikin, Zoellner, & Rizvi, 2013).

Soybean sprouts in Asia are generally prepared by soaking the seeds for about 4 to 5 hr followed by placing them in a dark growth chamber (sprouter) at room temperature and watering them several times per day; under most conditions, sprouts can be ready for harvest 5–7 days after germination (Ghani, Kulkarni, Song, Shannon, & Lee, 2016; Liu, 1997; Silva et al., 2013). Air and water temperature of 20 ℃ and relative humidity of 80% are recommended to produce good quality sprouts (Ghani et al., 2016; Lee et al., 2007; Liu, 1997; Shi et al. 2010). These sprouting conditions, a pH close to neutral and nutrient availability, are also favorable for the growth of microorganism, if present on the soybean seeds.

Fresh produce, such as sprouts, can be a vehicle for the transmission of several bacterial, protozoan, and viral pathogens capable of causing human illness (FDA, 1999). This is the reason why sprouts represent a special food safety concern (NACMCF, 1999). From 1998 to 2010, 33 outbreaks from seed and bean sprouts were reported in the United States (Dechet et al., 2014). The most popular foodborne diseases are those caused by *Escherichia coli* O157:H7, *Salmonella, Campylobacter,* and *Cryptosporidium* (Johnston, Moe, Moll, & Jaykus, 2005). Fungi also have the potential, less than bacteria, to cause food spoilage and pathogenesis. However, the information of mycological identification of fungi species isolated from soybean sprouts was very limited. Eighteen and seven species of fungi were isolated from spoiled *Vigna* spp. and soybean sprouts in Japan, respectively; and approximately 70% of the isolates were plant pathogens and at least 14 species were known to be seed‐borne (Sato et al., 2014). The fungus species isolated from soybean included some strains of *F. graminearum* that have already been reported to produce high concentrations of deoxynivalenol (DON) (Sato et al., 2014). DON is a mycotoxin produced by certain *Fusarium* species, and mycotoxins are compounds produced by fungi that can contaminate food and feed commodities during both pre‐ and postharvest and when ingested, it causes mycotoxicoses, which can have acute or toxic effects on human and animals (Milićević, Škrinjar, & Baltić, 2010; Sobrova et al., 2010).

In soybean sprout outbreaks, the seeds are the potential source of contamination, although poor sanitation and unhygienic practices at the sprout operation could also produce contamination (NACMCF, 1999). As a result, seed disinfection treatments were considered the major intervention in a multistep approach to reduce the risk of illness associated with contaminated sprouts. Calcium hypochlorite (20,000 ppm), the standard treatment for over a decade, and sodium hypochlorite solution (10,000–50,000 ppm) are widely used as disinfection treatments (Ding, Fu, & Smith, 2013; FDA, 1999). However, one of the main limitations of the current chlorine‐based washes was the possible hazards associated with production, transporting, and handling large amounts of chlorines, thus making organic acids as a potential chemical alternative to disinfect seeds and sprouts (Sikin et al., 2013). Treatments of acetic acid, lactic acid, and a combination of lactic/acetic acid followed by calcium hypochlorite treatment have shown to be effective on reducing *E. coli O*157:H7 populations (Lang, Ingham, & Ingham, 2000). However, chemical treatment effectiveness may be limited by inaccessibility to pathogens sheltered in scarified surfaces and the interior of the seeds (Ding et al., 2013). Physical treatments are more environmentally friendly and may have better penetration characteristics than chemical treatments (Ding et al., 2013). Physical treatments include heat, radiation, electric and magnetic fields, high pressure, and ionization produce changes in the physical conditions, which can interfere with microorganism life and cause death. Some physical disinfection treatments can be applied to foods without causing deterioration, unlike chemical treatments (Stanga, 2010). In addition, seed biological interventions also have shown to be effective to eliminate pathogen populations on seed and/or sprouts (Ding et al., 2013). Combination of chemical and physical treatments has also shown to be an effective strategy. However, identifying the optimal conditions might be challenging due to the complexity introduced by the application of several treatments, but once established they might provide the best control (Ding et al., 2013). Reported studies on disinfection treatments focused on eliminating bacteria such as *E. coli* and *Salmonella* due to the high risk they represented for human health*.* Therefore, additional studies of alternative seed disinfection treatments against other microorganisms, such as fungi, were needed to improve management strategies and sprout safety.

A successful seed treatment should reduce microbial pathogens while preserving seed viability, germination (95%), seed vigor, and the sensorial attributes of the final product (FDA, 2017; Sikin et al., 2013). Despite the fact that soybean sprouts are in high demand in the edible market due to their numerous health benefits (Ghani et al., 2016), limited information was available on fungus species present on soybean seeds used for sprouting and effective seed disinfection treatments. Therefore, soybean seeds from a commercial sprout soybean cultivar, MFS‐561, were evaluated for the presence of fungi, which allowed us to identify potential risks for food spoilage and pathogenesis in soybean sprout production in VA, USA. In addition, effectiveness of several seed disinfection treatments and their effect on important sprout quality traits were also evaluated to establish effective and practical sanitation procedures of soybean seeds.

## 2 MATERIALS AND METHODS

### 2.1 Materials

Montague Farms, Inc., provided composite seed samples of MFS‐561, a sprout commercial variety from three different growing regions: southern Virginia (SV), eastern Virginia (EV), and northeastern North Carolina (NC) in the United States. Seeds were produced by a network of growers who supply seeds to Montague Farm, Inc.

### 2.2 Fungal isolation from soybean sprout seeds

MFS‐561 seeds from SV, EV, and NC harvested in 2015 were used for fungal isolation and identification. In order to determine the fungal incidence, seed surfaces were rinsed with sterile water. Afterward, 10 seeds were placed in holes punched on 4% potato a dextrose agar (PDA) plate that is a relatively rich medium for growing a wide range of fungi. Three plates from each region were incubated at room temperature (20℃) for five days. Then, each fungal colony that grew on the seeds was isolated by cutting a small piece from the edge of the mycelial of each individual colony. The mycelial pieces were transferred to fresh PDA plates and incubated at 20℃ for 7 days. The diameter of each fungal colony was recorded. After the incubation period, a piece of the mycelial from each fungal colony was transferred to 250 ml Erlenmeyer flask containing potato dextrose broth (PD; Difco). In order to produce enough fresh fungal mycelium for molecular identification, liquid cultures were grown for 10–14 days on a Lab‐line Incubator Shaker Orbit (400 rpm) at room temperature before DNA extraction. Fungi colonies on PDA were characterized morphologically, and a total of 55 isolations from southern Virginia (23), northeastern North Carolina (13), and eastern Virginia (19) were sent for sequencing.

### 2.3 Molecular identification of fungal species by DNA extraction, PCR, and sequencing

For extraction of genomic DNA of fungal isolations, mycelium from axenic cultures grown in PD broth was ground to a fine powder in liquid N_2_ using a mortar and a pestle. Genomic DNA was extracted following the CTAB method as described by Gontia‐Mishra, Tripathi, and Tiwari (2014). DNA was eluted in 100 µl of distilled H_2_O and diluted to a concentration of 30 ng/µl prior to PCR. The complete ITS 1 and 2 regions, along with the short structural gene (5.8S), were amplified with ITS5 (5′‐GGAAGTAAAAGTCGTAACAAGG‐3′) and ITS4 (5′‐TCCTCCGCTTATTGATATGC‐3′) by PCR (White, Bruns, Lee, & Taylor, 1990). The ITS regions were chosen to identify fungal species, as they have been widely used as a DNA barcoding regions for molecular identification of fungi (Schoch et al., 2012). Amplifications were carried out in 25 µl reaction mixtures containing 15.39 µl of sterile water, 2.4 µl of 10× PCR buffer, 0.8 µl of 50 mM MgCl_2_, 0.25 µl of each primer (100 µM), 0.5 µl of 10 µM dNTPs, 1.26 µl of 10% DMSO, 0.4 µl of Taq DNA polymerase (Apex Taq, 5 U/µl), and 4 µl of DNA template (30 ng/µl). PCRs were repeated twice to obtain 50 µl of PCR product for each fungus isolated. PCRs were performed as follows: an initial denaturation for 5 min at 95°C, 40 thermal cycles (1 min at 94°C, 1 min at 55°C and 1 min at 72°C), and a final 10 min extension at 72°C. PCR products were run on 1% agarose gel to verify amplification. The amplified DNA (50 µl) was purified using QIAquick PCR Purification Kit following manufacturer's instructions (QIAGEN) and sequenced (Eurofins Genomics). The sequence data were searched with “Standard Nucleotide BLAST” in the NCBI website (https://blast.ncbi.nlm.nih.gov/Blast.cgi?PROGRAM=blastn&PAGE_TYPE=BlastSearch&LINK_LOC=blasthome) to identify the species; results were compared and confirmed based on the fungal colonies morphology on PDA.

### 2.4 Disinfection seed treatments

MFS‐561 seeds from SV, EV, and NC harvested in 2015 were used for testing seed disinfection treatments. Seed treatments included physical intervention methods of hot water at 60°C for 2 min and dry heat at 50℃ for 1 hr, and chemical intervention methods of calcium hypochlorite (2% w/v Ca [ClO] _2_) for 10 min, sodium hypochlorite (2% v/v NaOCl) for 10 min, acetic acid (5% v/v) for 2 min, and lactic acid (5% v/v) for 10 min. Commercial bleach with 8.25% NaOCl (Clorox), granulated calcium hypochlorite with 65% content of chlorine (Pfaltz & Bauer), 99% acetic acid (Fisher Chemical), and 10% lactic acid (RICCA Chemical Company) were diluted in sterile water for preparing the treatment solutions of calcium hypochlorite, sodium hypochlorite, acetic acid, and lactic acid, respectively. The pH of the treatment solutions was 11.97 for calcium hypochlorite, 8.7 for sodium hypochlorite, and 2.9 for acetic and lactic acid. Ten seeds from each growing region were placed in a 50‐ml beaker and covered with 40 ml of the respective disinfection solutions during the times specified above. For the physical interventions, ten seeds were placed in a strainer and submerged in the water bath for 2 min for the hot water treatment; ten seeds were placed in a container and put in the oven for 1 hr for the dry heat treatment; and temperatures were set at 60 and 50℃, respectively. After chemical and physical treatments, seeds were rinsed with sterile water for 1 min and placed in holes punched on 4% PDA (Potato dextrose agar) plates; each plate had 10 seeds. Plates were incubated at room temperature (20 ℃) for five days, and the number of infected seeds out of the ten initial seeds was recorded as the fungus incidence. Sterile water treatment was used as a control, and each treatment was repeated five times.

### 2.5 Effect of seed treatments on sprout traits

A total of four composite seed samples of MFS‐561 were used to study the effect of seed disinfection treatments on sprout traits. Seed source including growing region and year is provided in Table 1. A total of 350 unbroken and undamaged seeds of MFS‐561 from each composite sample were selected and grown in a bean sprouter (Cheong Si Ru, SC‐9000TS, made in Korea) at room temperature for five days. Before sprouting, seeds were treated with 2% calcium hypochlorite for 10 min, 5% acetic acid for 2 min, hot water (60℃) for 2 min, and sterile water for 1 min (control) as described previously; each treatment was repeated three times for one seed sample. Sprouting conditions and sprout trait evaluation were performed as described by Escamilla, Rosso, Strawn, and Zhang (2017). Sprout traits evaluated include percentage of high‐, average‐, and low‐quality sprouts, hypocotyl length and thickness, and sprout yield.

**Table 1:** Composite seed samples of MFS‐561 used to evaluate seed disinfection treatment effect on sprout traits

Sample ID^a^	Growing region	Year	Amount (g)
CS1	Southern Virginia	2015	453.6
CS2	Eastern Virginia	2015	453.6
CS3	Southern Virginia	2016	453.6
CS4	Northeastern North Carolina	2016	453.6

a CS, Composite seed sample.

### 2.6 Statistical analysis

Statistical analysis was computed in JMP statistical version 11.0 (SAS Institute). Prior to conducting statistical analyses, assumptions for two‐way analysis of variance model were checked for all response variables. The normality assumption was assessed by Shapiro–Wilk test and normal probability plots, while the assumption of homogeneity of variance was evaluated by residuals’ versus predicted values’ plots and plots of residuals by groups (McKillup 2012). Variables that violated the assumptions of normal distribution and equal variance were properly transformed and analyzed. All variables met assumptions after transformation. Seed disinfection treatment performances were evaluated under a two‐factor completely randomized design with seed treatments (6 treatments + control) and locations (SV, EV, and NC) as factors. A two‐factor completely randomized design with seed treatments (3 treatments + control) and composite seed sample (CS1, CS2, CS3, and CS4) as factors was used to assess the effect of seed disinfection treatments on sprout traits. The seed treatment effects on fungi incidence and sprout quality traits were analyzed using a two‐way analysis of variance (ANOVA). Tukey's HSD test was used to show the treatments that differed significantly at *p* = .05.

## 3 RESULTS

### 3.1 Fungi species isolated from MFS‐561, a commercial soybean sprout cultivar in VA, USA

A total of 55 isolates identified as seven species and six genera were obtained from MFS‐561 seeds across the three regions. Fungal species, source locations, and most representative accessions with high identity hit in each BLASTN search were listed in Table 2. The most common genera across three growing regions were *Alternaria* (29.1%)*, Diaphorte* (29.1%), and *Fusarium* (23.6%). While *Phoma* (1.8%), *Penicillium* (3.6%)*,* and *Cladosporium* (7.3%) were identified only on one or two locations. *Fusarium equiseti* (30.77%) was the most common species in NC followed by *Diaphorte longicolla* (23.1%) and *Penicillium citrinum* (14.4%). In SV, the most common species were *Alternaria alternata* and *Diaphorte* sp. with frequency of 34.8%. *A. alternata* (15.8%) and *D. longicolla* (15.8%) were also the most common species in EV together with *Fusarium* sp. (15.8%) (Table 3).

**Table 2:** Fungi isolated from seeds of a sprout soybean cultivar growth in southern Virginia, eastern Virginia, and northeastern North Carolina in 2015

Division^a^	Species	Isolation source	Location source^b^	Year	BLAST hit (accessions)^c^	Homology^d^
A	*Alternaria alternata*	Seeds	NC, SV, and EV	2015	KX783406.1	100/95
A	*Alternaria* sp.	Seeds	EV and SV	2015	KX878965.1	100/92
A	*Cladosporium cladosporioides*	Seeds	EV and SV	2015	KX258800.1	99/100
A	*Diaporthe longicolla*	Seeds	NC and EV	2015	KX878969.1	98/99
A	*Diaporthe* sp.	Seeds	NC, SV, and EV	2015	MF435146.1	90/97
A	*Fusarium chlamydosporum*	Seeds	EV	2015	KU516827.1	82/94
A	*Fusarium proliferatum*	Seeds	SV	2015	JQ957846.1	90/77
A	*Fusarium equiseti*	Seeds	EV and NC	2015	KX878922.1	93/95
A	*Fusarium* sp.	Seeds	EV and NC	2015	KU886151.1	97/93
A	*Penicillium citrinum*	Seeds	NC	2015	KX867539.1	99/99
A	*Phoma* sp.	Seeds	SV	2015	KM387394.1	90/80

a A: Ascomycota. Divisions of fungi species were found at Mycobank webpage.

b SV: Southern Virginia, EV: eastern Virginia, NC: northeastern North Carolina.

c Strains identification preserved at the DNA DataBank of Japan (DDBJ), the European Nucleotide Archive (ENA), and GenBank at NCBI.

d Identity (%)/query coverage (%).

**Table 3:** Relative frequency of fungal species by growing regions southern Virginia, eastern Virginia, and northeastern North Carolina

Species	% Frequency			
	NC^a^	SV^a^	EV^a^	Total^b^
*Alternaria alternata*	7.69	34.78	15.79	21.82
*Alternaria* sp.	0.00	8.70	10.53	7.27
*Cladosporium cladosporioides*	0.00	8.70	10.53	20.00
*Diaphorte longicolla*	23.08	0.00	15.79	10.91
*Diaphorte* spp.	7.69	34.78	5.26	18.18
*Fusarium Chlamydosporum*	0.00	0.00	10.53	3.64
*Fusarium equiseti*	30.77	0.00	10.53	10.91
*Fusarium proliferatum*	0.00	4.35	0.00	1.82
*Fusarium* spp*.*	7.69	0.00	15.79	7.27
*Penicillium citrinum*	15.38	0.00	0.00	3.64
*Phoma* spp*.*	0.00	4.35	0.00	1.82
Uncultured fungus	7.69	4.35	5.26	5.45

a The growing region names are abbreviated as follows: NC is northeastern North Carolina, SV is southern Virginia, and EV is eastern Virginia.

b Total frequency across all regions.

### 3.2 *Alternaria* sp. and *A. alternata*

**Figure 1:**
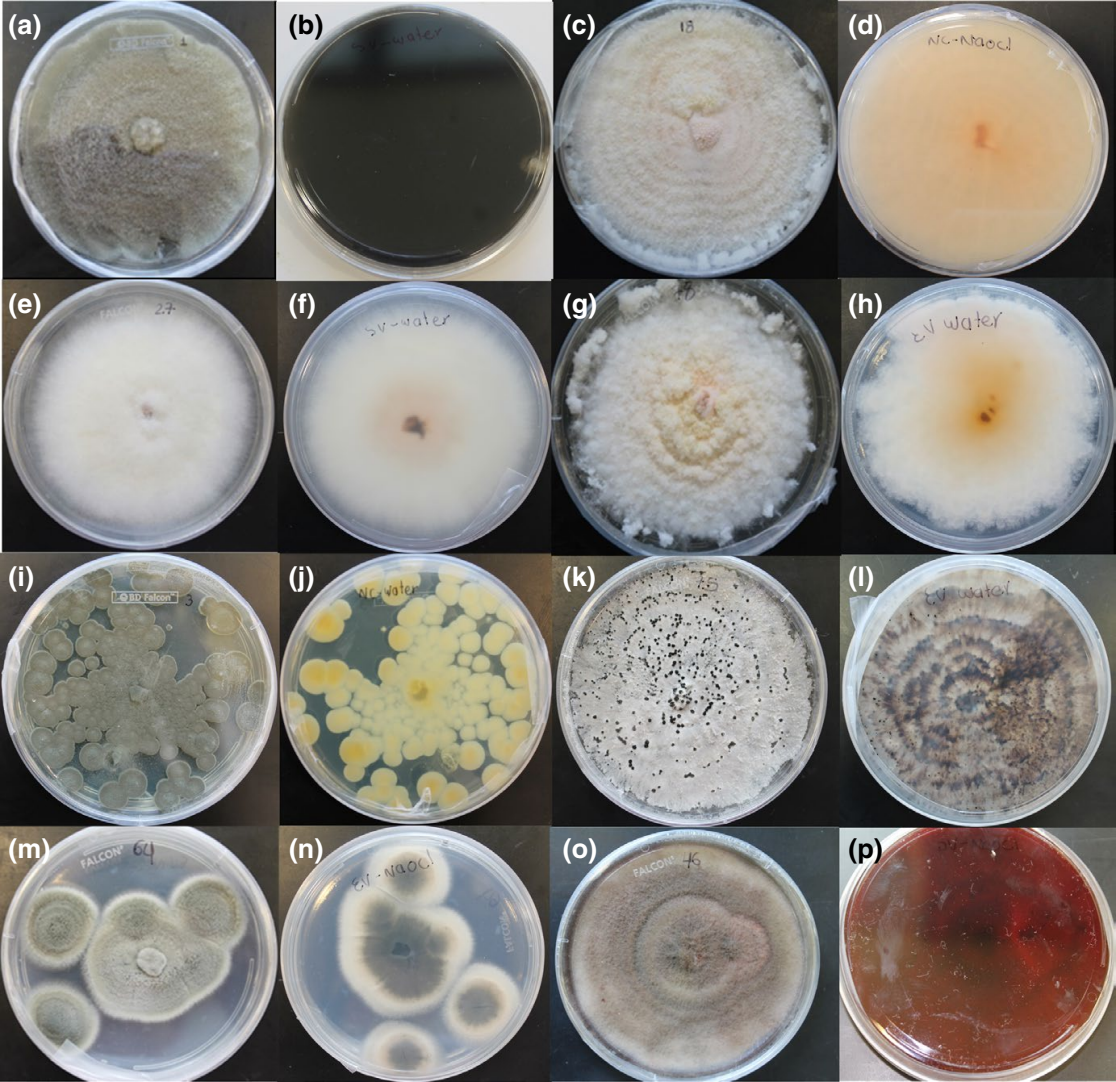
Fungi isolated from seeds of a commercial sprout cultivar in VA, the United States. (a) surface side of a colony on a PDA plate of *Alternaria alternata*, (b) reverse side of *A. alternata* colony, (c) surface side of a colony on a PDA plate of *Fusarium equiseti*, (d) reverse side of *F. equiseti* colony, (e) surface side of a colony on PDA plate of *Fusarium proliferatum*, (f) reverse side of *F. proliferatum* colony, (g) surface side of a colony on PDA plate of *Fusarium chlamydosporum,* (h) reverse side of *F. chlamydosporum* colony, (i) surface side of a colony on a PDA plate of *Penicillium citrinum,* (j) reverse side of *P. citrinum* colony, (k) surface side of a colony on a PDA plate of *Diaphorte longicolla,* (l) reverse side of *D. longicolla* colony, (m) surface side of a colony on PDA plate of *Cladosporium cladosporioides,* (n) reverse side of *C. cladosporioides* colony, (o) surface side of a colony on PDA plate of *Phoma* sp., (p) reverse side of *Phoma* sp. colony

*Alternaria alternata* was isolated from NC, SV, and EV with a frequency of 7.7%, 34.8%, and 15.8%, respectively. Several isolations were identified as *Alternaria* sp. on SV (8.7%) and EV (10.53%) seeds when searching on NCBI databases (Table 3). When growing in PDA plates, *A. alternata* species were characterized by colonies that could either have 50–70 mm diameter or cover the whole Petri dish with a gray brown mycelium and a brown to nearly black color on the reverse side (Pitt & Hocking, 2009). In this study, colonies of *A. alternata* and *Alternaria* sp. had a gray brown mycelium covering the whole plate and a black color on the reverse (Figure 1a‐b). There were not noticeable differences on colony morphology among *A. alternata* and *Alternaria* spp. isolations.

### 3.3 *Diaphorte* sp. and *D. longicolla*

*Diaporthe* sp. represented a 7.7%, 34.8%, and 5.3% from the total isolations of NC, SV, and EV, respectively; and *D. longicolla* represented a 23.1% and 15.8% from the total isolations of NC and EV, respectively (Table 3). *Diaporthe longicolla* (anamorph = *P.* longicolla) colonies on PDA were described for the first time by Hobbs, Schmitthenner, and Kuter (1985) as floccose, dense, and white with occasional greenish‐yellow areas and colorless in the reverse with large black stomata; similar characteristics were observed on *Diaphorte* sp. and *D. longicolla* isolations from this study (Figure 1k‐l). There were not significant morphological differences among colonies of *D. longicolla* and *Diaphorte* sp.

### 3.4 *Fusarium* sp., *F. proliferatum*; *F. equiseti*, and *F. chlamydosporum*

*Fusarium* species were found in significant frequencies on soybean seeds of NC (38.46%) and EV (26.32%). A percentage of 7.7 from NC and 15.8 from EV of the total isolations were identified at genus level as *Fusarium* sp. when searching on NCBI databases. Three *Fusarium* species were isolated from soybean seeds. One was *F. chlamydosporum* detected at low frequency in EV (10.53%) (Table 3). Its colonies presented a powdery appearance (felty mycelium) from profuse microconidial production on PDA, with pale salmon color and paler margins as described by Pitt and Hocking (2009) (Figure 1g‐h). Other was *F. proliferatum* found at low frequency (4.35%) in SV (Table 3). Its colonies were floccose with a pale orange to white mycelium and a light orange in the reverse in accordance with Pitt and Hocking (2009) (Figure 1e‐f). Lastly, *F. equisete* isolated in high frequency (30.77%) on soybean seeds from NC and in low frequency (10.53%) on seeds from EV (Table 3). On PDA, *F. equiseti* colonies had floccose mycelium with white to pale salmon color that became brown with age, a central mass of orange sporodochia sometimes surrounded by poorly defined sporodochial rings and a pale salmon color on the reverse often flecked with brown (Pitt & Hocking, 2009). Colonies of *F. equiseti* isolated in this study showed several of these characteristics (Figure 1c‐d).

### 3.5 Cladosporium cladosporioides

*Cladosporium cladosporioides* was detected on soybean seeds from SV (8.7%) and EV (10.5%) at low frequencies (Table 3). Its colonies were low and dense, lightly wrinkled surface, lightly floccose, and olive color with a dark gray on the reverse as described by Pitt and Hocking (2009) (Figure 1m‐n).

### 3.6 Penicillium citrinum

*Penicillium citrinum* was only isolated from NC soybean seeds representing 15.4% of total isolations from North Carolina (Table 3). Colonies of *P. citrinum* are floccose with a yellow brown or olive color, white mycelium in peripheral areas, radially sulcate, and dull brown color on the reverse (Pitt & Hocking, 2009). In this study, *P. citrinum* colonies exhibited similar characteristics but with a dull yellowish brown color in the reverse (Figure 1i‐j).

### 3.7 *Phoma* sp.

A low percentage (4.3%) of isolations from SV was identified as *Phoma* sp. (Table 3). Its colonies have a color that varies from dark to black, olive‐gray, or grayish‐brown, and a diffusible pigment either reddish or brown is sometimes released into the media. The colonies do not wrinkle but they may form concentric circles with darker shades of color in the center (Sciortino, 2017). In this study, colonies of *Phoma* sp. had an olive‐gray color with a reddish pigment clearly visible in the front and reverse of the PDA plate and concentric circles (Figure 1o‐p).

### 3.8 Disinfection seed treatments

**Figure 2:**
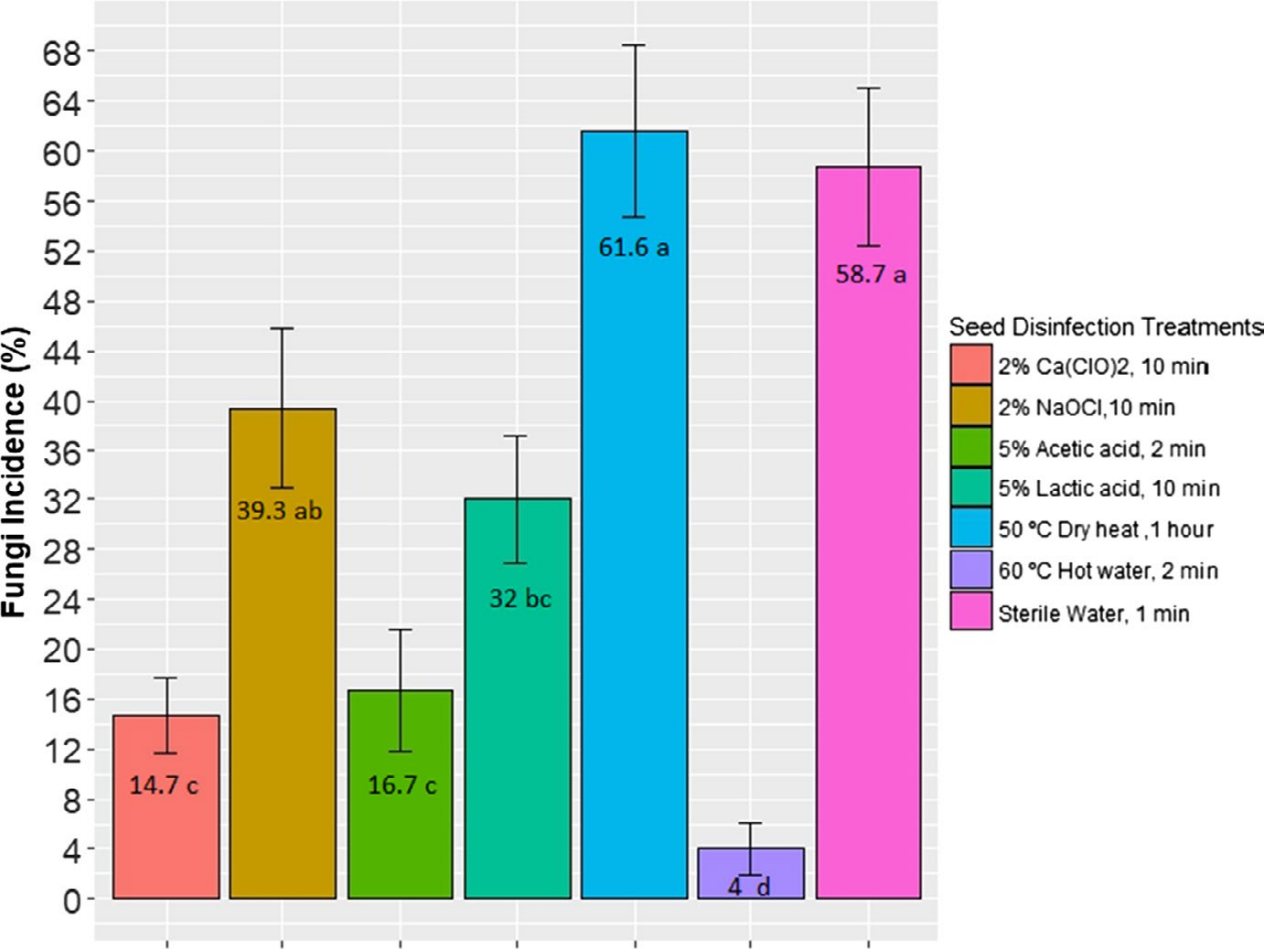
Average fungus incidence on soybean seeds by seed treatments across three different seed production regions. Different letters represent significant differences at *p* ≤ .05 level

Seed disinfection treatments had a significant effect on fungi incidence, and there were not significant differences among growing regions (Table 4). Nontreated seeds had a fungi incidence of 58.7%. The most effective treatments were hot water at 60 ℃ for 2 min, 2% calcium hypochlorite for 10 min, and 5% acetic acid for 2 min with 4%, 14.7%, and 16.7% of fungi incidence, respectively (Figure 2). The lactic acid treatment reduced significantly fungi incidence compared with the control; however, seed fungal contamination was still high (32%). Dry heat and sodium hypochlorite treatments were the most ineffective on reducing fungal populations with mold percentages of 61.6% and 39.3%, respectively. Therefore, calcium hypochlorite, acetic acid, and hot water were selected as potential seed disinfection treatments and their effects on sprout quality traits were evaluated.

**Table 4:** Analysis of variance results of seed disinfection treatments across three growing regions: NC is northeastern North Carolina, SV is southern Virginia, and EV is eastern Virginia

Source	*df* a	Sum of square	*F* Ratio	*p*‐value
Seed treatment (ST)	6	533.58	23.91	<.0001*
Growing Region (GR)	2	18.82	2.53	.0857
ST*GR	12	73.63	1.65	.0934

a DF is degrees of freedom.

* Significant at *p* ≤ .05 level.

### 3.9 Effect of decontamination treatments on sprout traits

Seed treatments (ST) had a significant effect on sprout quality traits and yield, but not for hypocotyl length and thickness. Sprout quality traits and hypocotyl length were significantly different across composite seed (CS) samples, because sprout traits are highly determined by the environments where seeds are grown (Escamilla et al., 2017). ST and CS interaction affected significantly sprout quality (Table 5), likely associated with the specific fungal species found on each composite seed sample. Calcium hypochlorite and acetic acid treatments did not produce any adverse effect on sprout traits compared with the control. Seeds treated with calcium hypochlorite, acetic acid, and sterile water had high‐quality sprout (HQS) % and average quality (AVQ) % of 40.21% and 24.05%, 37.52% and 25.41%, and 38.81% and 21.5%, respectively. Low‐quality sprout (LQS) % and sprout yield ranged from 35.7% to 39.7% and 3.05 to 4.07 g/100 g seeds, respectively (Table 6). Similar results were found in a previous sprout trait study with HQS% and AQS% that ranged from 33.3% to 62.7% and 28 to 48.2%, respectively (Escamilla et al., 2017). Sprouts in this study had a lower sprout yield and higher LQS% than those previously reported by Escamilla et al. (2017), where they ranged from 5.24 to 7.01 g/100 g seeds and 7.7 to 29.7%, respectively. It suggested a lower seed quality in this study, which resulted in lower quality sprouts and sprout yield. These differences were likely caused by the environments because seed quality largely depended upon environmental conditions. On the contrary, hot water treatment produced sprouts with lower HQS% (6.76) and sprout yield (3.05) compared with the control and other treatments, while producing sprouts with higher AQS% and LQS% (Table 6), thus affecting negatively sprout quality and seed germination.

**Table 5:** Analysis of variance of sprout traits for composite soybean seed samples from southern Virginia 2015 and 2016, and eastern Virginia and northeastern North Carolina 2015 treated with seven seed disinfection treatments

Source	DF^a^	Sprout Traits^b^					
		HQS%	AQS%	LQS%	Hlght	Hthk	Syld
Treatment (T)	3	<0.0001*	<0.0001*	<0.0001*	0.7577	0.404	0.0400*
Composite seed sample (CS)	3	<0.0001*	<0.0001*	0.0006*	<0.0001*	0.0979	0.6193
T*CS	9	0.0029*	<0.0001*	0.0013	0.3851	0.4951	0.2975

a *df* is degrees of freedom.

b The seed and sprout trait names are abbreviated as follows: HQS% is high‐quality sprout percentage, AQS% is average quality sprout percentage, LQS% is low‐quality sprout percentage, Hlgth is hypocotyl length, Hthk is hypocotyl thickness, and Syld is sprout yield.

* Significant at *p* ≤ .05 level.

**Table 6:** Mean of sprout quality traits of seed disinfection treatments

Treatments	HQS%^a^		AQS%^a^		LQS%^a^		Syld (g/g seed)^a^		Hlght (cm)^a^		Hthk (mm)^a^	
	Mean	SE^b^	Mean	SE	Mean	SE	Mean	SE	Mean	SE	Mean	SE
Ca(ClO)2 2%, 10 min	40.21 a *	2.88	24.05 b	2.45	35.74 b	2.74	4.07 a	0.23	9.64 a	0.43	1.74 a	0.05
Acetic acid 5%, 2 min	37.52 a	3.02	25.41 b	2.32	37.07 b	1.88	3.74 ab	0.15	9.20 a	0.54	1.71 a	0.02
Hot water 60°C, 2 min	6.76 b	1.20	42.43 a	3.6	50.81 a	3.65	3.05 b	0.22	9.46 a	0.37	1.73 a	0.01
Sterile Water 1 min	38.81 a	4.27	21.50 b	2.06	39.69 b	3.37	3.46 ab	0.35	9.20 a	0.55	1.74 a	0.05

a The sprout trait names are abbreviated as follows: HQS% is high‐quality sprout percentage, AQS% is average quality sprout percentage, LQS% is low‐quality sprout percentage, Hlgth is hypocotyl length, Hthk is hypocotyl thickness, Syld is sprout yield, and DF is degrees of freedom.

b SE is standard error.

superscript letters means with the same letter are not significantly different from each other (P.0.05).

*Significant at p<.05 level.

## 4 DISCUSSION

### 4.1 Fungi species isolated from MFS‐561, a soybean sprout cultivar in VA, USA

Seven species were isolated from *Glycine max* (Merr. L.) and identified as ascomycetous fungi, where the most common genera across three regions were *Alternaria* and *Diaphorte* followed by *Fusarium*. *Diaphorte* (anamorph = *Phomopsis*) species are associated with several soybean diseases, where *Diaporthe longicolla* (anamorph = *Phomopsis longicolla*) is the primarily cause of seed decay of soybeans and it is the major cause of poor‐quality seed (Darwish, Li, Matthews, & Alkharouf, 2016; Li, Hartman, & Boykin, 2010; Mengistu, Castlebury, Morel, Ray, & Smith, 2014; Santos, Vrandecic, Cosic, Duvnjak, & Phillips, 2011; Sun et al., 2013). *Fusarium* species are also associated with several plant diseases such as vascular wilts, root and stem rots among others (Pitt & Hocking, 2009). Therefore, the presence of field fungi such us *Fusarium* and *Diaphorte* species was expected because of its high pathogenicity to soybeans. Most *Alternaria* species are saprophytes, others well known postharvest pathogens, and opportunistic plant pathogens causing several diseases on important agronomic crops (Thomma, 2003). Previously, *A. alternata* was detected at high frequencies in soybean seeds in Argentina (Broggi, González, Resnik, & Pacin, 2007; Oviedo, Ramirez, Barros, & Chulze, 2009). Thus, *Alternaria* species can be found either as storage or as field fungi.

The less frequent species were *C. cladosporioides, P. citrinum,* and *Phoma* sp. *C. cladosporioides* and *P citrinum* have been previously isolated from soybean seeds and are considered a significant storage fungus of soybean seeds (Kim, Kim, Kwon, Lee, & Hong, 2013; Pitt et al.., 1994). Lastly, *Phoma* sp. is rarely observed pathogen, cosmopolitan, ubiquitous species on diseased and dead plant materials that is defined frequently as opportunistic parasite. *Phoma* sp. has been isolated from spots on leaves, pods, and seeds of soybeans, and other crop seeds (Kövics, Sándor, Rai, & Irinyi, 2014). It has also been found on spoiled sprouts of mung bean and soybeans (Sato et al., 2014). Thus, the species isolated from sprout soybean seeds included field and storage fungi.

In this study, most of the species isolated have been previously associated with mycotoxin production except *D. longicolla* and *C. cladosporioides.* The most widely recognized mycotoxin producers are *Fusarium* species, *A. alternata,* and *P. citrinum.* Some *Alternaria* spp. are of clinical importance because they produce toxic secondary metabolites such as alternariol, altenusin, and tenuazonic acid among others, and some of these mycotoxins were associated with the development of cancer in mammals, where *A. Alternata* particularly is gaining distinction as an emerging human pathogen (Bottalico & Logrieco, 1988; de Souza, Mithofer, Daolio, Schneider, & Rodrigues‐Filho, 2013; Thomma, 2003). *Fusarium* is one of the three major fungal genera producing toxins in infected plants and/or in plant products, where a high number of compounds (>50) are known to be produced by *Fusarium* species and some are highly toxic (Ding et al., 2013; Pitt & Hocking, 2009). Three *Fusarium* species were isolated from sprout soybean seeds in this study *F. equiseti*, *F. chlamydosporum,* and *F. proliferatum*. These *Fusarium* species are well known for producing different mycotoxins of type A and B trichothecenes, zearalenone, moniliformin, beauvericin, fusarochromanone, fusarin, and related compounds (Desjardins, 2006; Desjardins et al., 2000; Hestbjerg, Nielsen, Thrane, & Elmholt, 2002; Ivic, 2014; Ivic, Domijan, Peraica, Milicevic, & Cvjetkovic, 2009; Kosiak, Holst‐Jensen, Rundberget, Gonzalez Jaen, & Torp, 2005; Langseth, 1998; Logrieco et al., 1998; Marasas, Thiel, Rabie, Nelson, & Toussoun, 1986; Park & Chu, 1993; Ross et al., 1990). *Penicillium citrinum* is also known for producing the mycotoxin citrinin and cellulose digesting enzymes like cellulose and endoglucanase (Khan et al., 2008). Tenuazonic acid, viridactol, alternariol, and alternariol monomethyl ether production by *Phoma* sp. has been previously reported (Mousa et al., 2015; Steyn & Rabie, 1976). However, there is limited information about mycotoxin production by *Phoma* sp. and the significance of these toxins.

Presence of mycotoxins in food, seeds, or animal feed depends on many factors such as environmental conditions (storage conditions), fungal strain specificity, strain variation, and instability of toxigenic properties. Environmental conditions that favor fungus growth not necessarily favor mycotoxin production (Langseth, 1998; Oviedo et al., 2009; Zain, 2011). Identification of the specific mycotoxin is necessary to make a good diagnosis, which is generally done by liquid chromatography of extracts from the fugal source and/or food (Bennett & Klich, 2003; Plumlee & Galey, 1994). For that reason, the identification of *A. alternata, Fusarium* species and *P. citrinum* on sprout soybean seeds does not necessarily mean mycotoxin production and/or sprout contamination even when the species has already been associated with mycotoxins. However, the frequent appearance of fungal species that could produce mycotoxins represents a potential risk of multiple mycotoxin contamination on soybean seeds and possibly on soybean sprouts. Besides, fungi presence on soybean seeds could also reduce sprout quality and produce economic losses for soybean sprout growers and industry. Therefore, good seed storage conditions and adequate soybean seed disinfection treatments are required to eliminate seed‐borne fungi on soybean seeds among all other approaches at different critical control points that are being developed to avoid mycotoxin contamination in food chains. Further studies are needed to evaluate the toxigenic ability of fungal species associated with soybean seeds in Virginia, U.S.A, optimal conditions for growth and mycotoxin production, and the occurrence of their toxins in soybeans sprouts, if produced.

### 4.2 Disinfection seed treatments

Hot water at 60℃ for 2 min was the most effective treatment for reducing fungal soybean seed contamination. Previous reports showed that a wide range of hot water treatments at different temperatures and exposure times have successfully disinfected seeds of radish, alfalfa, and mung beans contaminated with *E. coli* and *Salmonella* with reductions of more than 5log CFU/g (Enomoto, Takizawa, Ishikawa, & Suzuki, 2002; Jaquette, Beuchat, & Mahon, 1996; Pao, Kalantari, & Khalid, 2008; Weiss & Hammes, 2005). These treatments consisted of different combinations of water temperatures ranging from 57 to 100℃ and exposure time to the treatment from seconds to 5 min (Jaquette et al., 1996; Pao et al., 2008). Hot water treatment has been widely used against bacteria such as *Salmonella* and *E. coli* because they are the most common causes of foodborne illness. Our results showed that hot water treatment was also effective on reducing field and storage fungi incidence on soybean seeds. Further studies at several temperatures and times might be needed to identify the conditions (temperature and time) that allow total elimination of fungi from soybean seeds.

Calcium hypochlorite (20,000 ppm for 10 min) was the second most effective seed treatment. Its effectiveness in reducing microbial pathogens has been evaluated in the past (Damron et al., 2005; Ding et al., 2013). The U.S. Food and Drug Administration (FDA) has recommended widely pregermination treatments with 20,000 ppm Ca(OCl)_2_ in sprout production (Christopher et al., 2003; Jaquette et al., 1996). Chlorine concentrations of 20,000 and 2,000 ppm have shown to be effective controlling *E. coli* and *Salmonella*, respectively, in alfalfa seeds (Jaquette et al., 1996; Lang et al., 2000). The effect of sodium and calcium hypochlorite has been studied mainly on bacteria as *E. coli* and *Salmonella* due to their importance as human pathogens. In this study, 2% calcium hypochlorite was more effective on reducing fungi incidence than 2% sodium hypochlorite treatment with 39.3% fungi incidence (Figure 2). Leonardo et al. (2016) found that when diluted at the same concentration, chlorine was expected to have a higher content in calcium hypochlorite solutions than in sodium hypochlorite solutions, making calcium hypochlorite a more effective seed treatment. In addition, chlorine solutions were more unstable at acidic pH with a limit temperature of 50 ℃ to prevent risk of corrosion (Stanga, 2010). In this study, the pH of calcium hypochlorite solution (11.97) was higher than the pH of sodium hypochlorite solution (8.7). Therefore, higher chlorine content and a more stable solution might be the reason why calcium hypochlorite treatment had high effectiveness as seed disinfectant. In general, low concentrations (1%–5%) of sodium hypochlorite are used for eliminating seed surface fungi but not internal fungi (Sauer & Burroughs, 1986). Therefore, higher concentrations of sodium hypochlorite may eliminate pathogen populations in the seeds.

The third most effective treatment was 5% acetic acid followed by 5% lactic acid with seed fungi incidence of 16.8 and 32%, respectively (Figure 2). Lactic and acetic acid treatments at concentrations of 5% for 2 and up to 10 min have successfully controlled bacteria populations of *E. coli* and *Salmonella* on alfalfa and radish seeds with reduction of 2–5 log CFU/g. However, these treatments did not prevent regrowth of surviving *E. coli* (Lang et al., 2000). Solutions of 5% acetic acid eliminated *Salmonella* from alfalfa and mung bean sprouts after 4 and 16 hr, respectively, while solutions of 2% acetic acid eliminated *Salmonella* after 24 and 48 hr for alfalfa and mung bean, respectively (Pao et al., 2008). Thus, higher concentration of acetic acid required shorter time to eliminate pathogen populations. In this study, seeds were treated with acetic acid for 2 min because longer times showed to affect adversely seed viability when observed on PDA plates. As a result, other combinations of exposure times and acetic/lactic acid concentrations might increase acetic and lactic acid effectiveness on reducing fungal populations on soybean seeds. The antibacterial activity of acetic acid has been proven in several studies (Halstead et al., 2015; Lang et al., 2000; Nei, Latiful, Enomoto, Inatsu, & Kawamoto, 2011; Pao et al., 2008). However, its antifungal activity has not been widely explored in the sprout industry with few reports (Cabo, Braber, & Koenraad, 2002). In this study, acetic acid treatment and lactic acid treatment significantly reduced fungi incidence on soybean seeds compared with control treatment, where a higher reduction in fungal population was achieved by acetic acid, being a potential seed disinfection treatment for sprout soybean seeds.

Dry heat at 50℃ for 1 hr was the most ineffective seed treatment probably because the temperature and/or exposure time were not enough for killing fungal populations. Similar treatment conditions, dry heat at 50 ℃ for 1 and 17 hr, have shown to be effective in eliminating *E. coli* and other pathogen populations from alfalfa, radish, mung bean, and broccoli seeds when combined with irradiation and chemical sanitizers, respectively (Bari, Nazuka, Sabina, Todoriki, & Isshiki, 2003; Bari, Nei, Enomoto, Todoriki, & Kawamoto, 2009b). In addition, it showed that a wrong procedure of disinfection by heating could result in higher fungi growth because temperature could have a positive effect on spores and make them sprout (Stanga, 2010). Exposure time and temperature to adequately sterilize seeds depend on pathogen populations (marginal growth temperatures) and seed type. Therefore, determination of marginal growth temperatures of fungal populations present on soybean seeds and the effect of high temperatures on soybean seed germination will allow establishing more effective dry heat treatments. The effectiveness of these seed treatments was also determined by the composite seed sample which can be directly associated with the type of fungal populations found on each seed sample. Further studies are needed to identify the effectiveness of these treatments on specific fungi species.

Consequently, a reduction of 93.2%, 74.9%, and 71.6% from the initial fungal levels can be achieved by treating soybean seeds with hot water at 60 ℃ for 2 min, 5% calcium hypochlorite for 10 min, and 5% acetic acid for 2 min, respectively. However, seed treatments could have negative effects on sprout quality. For this reason, it was important to evaluate their effect on sprout traits before making a seed treatment recommendation.

### 4.3 Effect of decontamination treatments on sprout traits

Calcium hypochlorite and acetic acid treatments did not produce any adverse effect on sprout traits compared with the control. Calcium hypochlorite and acetic acid treatment effects on seed germination have been reported previously in other plants. A study on alfalfa seeds treated with acetic acid, calcium hypochlorite, and a combination of both showed that germination of seeds was not adversely affected by any of the treatments obtaining germinations higher than 90% (Lang et al., 2000). Similarly, a treatment of 8.7% of acetic acid at 55℃ for 2–3 hr did not affect germination rates of alfalfa and radish seeds (Nei et al., 2011). Treatments of up to 5,000 ppm Ca(OCl)_2_ have not shown negative effects on germination rate and seed viability of alfalfa seeds (Damron et al., 2005; Ding et al., 2013; Jaquette et al., 1996). However, other studies showed that treatments of 200 ppm of active chlorine for 5 min reduced germination rate of alfalfa, broccoli, kohlrabi, kyona, mustard, red kohlrabi, red young radish, and violet radish when combined with dry heat (80℃ for 24 hr) (Choi, Beuchat, Kim, & Ryu, 2016). Seed treatments of cowpea seeds with 5% acetic acid for 45 min also significantly deteriorated seed germination (Singh, Chandra, Agrawal, & Babu, 2005). Therefore, seed tolerance to chlorine and acetic acid varied among crops, exposure time, and concentrations. As many studies showed that calcium hypochlorite and acetic acid treatment has not negative effect on seed germination, treatments of 2% calcium hypochlorite for 10 min and 5% acetic acid for 2 min were also recommended as soybean seed disinfectants in this study because they significantly reduced fungal populations on soybean seeds without affecting seed germination and sprout quality traits.

Despite this study showed that hot water was the best disinfectant seed treatment, hot water treatment significantly reduced sprouts quality and seed germination. It is well known that high and fluctuant temperatures speed up seed deterioration rates reducing seed germination and viability (Mbofung, Goggi, Leandro, & Mullen, 2013). Similarly, hot water treatments (85℃ for 9 s) on alfalfa seed reduced germination (73%) and yield (78.4%) compared with the control (Enomoto et al., 2002). However, some other studies with dry heat at several temperature and times have shown to be effective as seed disinfectants without adverse effects on germination rates (Bari, Sugiyama, & Kawamoto, 2009a; Jaquette et al., 1996). Hypocotyl length and thickness were not adversely affected by any of the treatments. As a result, despite its high effectiveness on reducing fungal populations hot water treatment at 60℃ for 2 min was not recommended for soybean seeds because it adversely affected germination rate and sprout quality by producing more sprouts with low and average quality. Further studies, exploring other temperatures and shorter times, might produce satisfactory results in disinfecting soybean seeds without compromising seed viability and sprout quality.

## 5 CONCLUSION

Most fungal species isolated in this study were associated with mycotoxin production, especially *Fusarium* spp., *P. citrinum,* and *A. alternata.* But their presence did not necessarily mean sprout and seed contamination by mycotoxins. In order to make a complete diagnosis, the specific mycotoxin must be detected from the fungal source and/or food. Further studies to evaluate the toxicological risk of isolated fungal strains will allow determining the actual risk that these species represent in sprout production in Virginia. Moreover, fungal contamination and frequent appearance indicated a potential risk for multiple mycotoxin contaminations of soybean seeds and sprouts, which could produce significant economic losses for soybean sprout growers and sprout industry. A significant reduction on initial levels of fungal populations could be achieved by treating soybean seeds with hot water, calcium hypochlorite, and acetic acid, respectively. However, seeds treated with hot water had a lower germination rate and poor sprout quality. As a result, seed disinfection treatments of 2% calcium hypochlorite for 10 min and 5% acetic acid for 2 min were recommended as potential seed disinfection treatments for soybean seeds because they reduced fungi incidence without reducing seed germination and sprout quality. None of the treatments completely eliminated the fungal populations, so optimization of relevant treatment parameters may be needed (temperature, time, and dosage) to achieve a higher control. Exploring other temperature types and exposure times for hot water treatment might produce satisfactory results in disinfecting soybean seeds without reducing seed germination. Antimicrobial seed treatments are specific for seed type and microbe, and they must be evaluated for the specific seed type under normal production conditions. Thus, calcium hypochlorite and acetic acid treatment were recommended specifically for soybean seeds to control fungal populations.

A complete elimination of microbial contamination by seed disinfection treatments in sprout production is very unlikely. Food contamination is possible even if seed treatments are successful because treated pathogens may survive. Thus, seed disinfection must be implemented together with other preventative strategies such as good agricultural practices (seed cleaning, seed storage, and handling), good manufacturing practices, and hazard analysis.

## CONFLICT OF INTEREST

The authors declare no conflict of interest.

## ETHICAL APPROVAL

The study conforms to the Declaration of Helsinki, USA, and/or European Medicines Agency Guidelines for human subjects. The protocols and procedures in this study were ethically reviewed and approved by Ethical Committee of Tochigi Prefectural Institute of Public Health and Environmental Science (approval numbers: H30‐000731). This work does not involve any animal or human testing.
